# Long-term management of Fontan patients: The importance of a multidisciplinary approach

**DOI:** 10.3389/fped.2022.886208

**Published:** 2022-08-25

**Authors:** Diletta Martino, Caterina Rizzardi, Serena Vigezzi, Chiara Guariento, Giulia Sturniolo, Francesca Tesser, Giovanni di Salvo

**Affiliations:** ^1^Pediatric Unit, Department for Women's and Children's Health, University Hospital of Padova, Padua, Italy; ^2^Pediatric and Congenital Cardiology Unit, Department for Women's and Children's Health, University Hospital of Padova, Padua, Italy

**Keywords:** Fontan, failure Fontan, complications, Fontan circuit, multidisciplinary

## Abstract

The Fontan operation is a palliative procedure that leads to increased survival of patients with a functional single ventricle (SV). Starting from 1967 when the first operation was performed by Francis Fontan, more and more patients have reached adulthood. Furthermore, it is expected that in the next 20 years, the population with Fontan circulation will reach 150,000 subjects. The absence of right ventricular propulsion and the inability to improve cardiac output because of the low cardiac reserve are the main issues with the Fontan circulation; however, potential complications may also involve multiple organ systems, such as the liver, lungs, brain, bones, and the lymphatic system. As these patients were initially managed mainly by pediatric cardiologists, it was important to assure the appropriate transition to adult care with the involvement of a multidisciplinary team, including adult congenital cardiologists and multiple subspecialists, many of whom are neither yet familiar with the pathophysiology nor the end-organ consequences of the Fontan circulation. Therefore, the aim of our work was to collect all the best available evidence on Fontan's complications management to provide “simple and immediate” information sources for practitioners looking for state of the art evidence to guide their decision-making and work practices. Moreover, we suggest a model of follow-up of patients with Fontan based on a patient-centered multidisciplinary approach.

## Introduction

Many complex cardiac malformations are characterized (functionally or anatomically) by the existence of only one ventricle that must sustain both pulmonary and systemic circulations, which are therefore not connected in series but connected in parallel. Such a circuit has two major disadvantages: arterial desaturation and a chronic volume overload to the single ventricle (SV) (1, 2). The Fontan operation is the palliative procedure of choice when a biventricular reconstruction is not feasible in cases, such as tricuspid atresia, double-outlet right ventricle, some types of pulmonary atresia, and atrioventricular septum defects with unbalanced ventricles (2–4). A cavopulmonary shunt is manufactured to connect the systemic venous return to the pulmonary arteries without the interposition of an adequate ventricle.

The consequences of these changes are numerous, which are as follows: increment in systemic venous pressure, decreased cardiac output, non-pulsatile hepatic congestion, and high pulmonary artery pressure. These features have an impact on both visceral organs and lymphatic drainage, causing multiorgan complications that require lifelong multidisciplinary management (4, 5). Despite the high morbidity and mortality, up to 70,000 people with Fontan-type palliation are alive worldwide, and the population is expected to double in the next 20 years, therefore, requiring appropriate transition to adult care (6).

Despite the existence of international and European guidelines (3, 7), the precise timing and panel of tests for the surveillance strategy in patients with Fontan is not universally defined and up until now in many countries, the follow-up is mostly based on the single centers' expertise without uniform application of diagnostic methods to screen for relevant outcomes (5). This article aims to review the state of the art of complications of Fontan palliation and to provide an example of a multidisciplinary approach, based on our center protocol and according to international guidelines, to guide clinicians in the management of these patients in work practice.

## Materials and methods

To write this article, we reviewed the literature to examine the state of the art of standardized programs for patients with Fontan. To select the works, we searched the major electronic databases (MEDLINE, Embase, Web of Science, and Scopus) using the terms “Fontan complications,” “Failure Fontan,” “single ventricle circulation,” “complications,” and “Fontan pathophysiology.” We selected only articles written in English without restriction based on the year of publication. Finally, we performed a selection considering only articles describing Fontan complications at least after 6 months of circulation compilation. We retrieved and assessed for eligibility all potentially relevant titles and abstracts.

## Complication of Fontan palliation

### Heart failure

Heart failure (HF) can result from systolic or diastolic ventricular dysfunction, and it occurs in 40%−60% of patients with Fontan (8). In the pediatric population, the systolic dysfunction is the most frequently identified, whereas diastolic dysfunction manifestations increase with the duration of follow-up (9). After the Fontan palliation, the SV evolves from being volume overloaded and overstretched to being overgrown in the face of a newly low preload. This deprivation in volume can lead to a progressive decline in cardiac output known as “disuse hypofunction” (1).

Other factors contributing to the development of HF include arrhythmias, increased neurohormonal activation, older age at repair, prolonged cyanosis, myocardial perfusion abnormalities, and atrio-ventricular valve (AVV) regurgitation (10). In particular, AVV failure occurs in nearly one-third of all patients by 30 years of age, more frequently affecting common AVV or tricuspid valves (2, 11). Clinical manifestations are similar to those of biventricular HF; cyanosis may develop due to veno-venous collaterals or in those patients with a fenestration between the intra-cardiac conduit and the pulmonary atrium, while the non-pulsatile flow to the lower lung segments may cause ventilation-perfusion mismatch and subsequent hypoxia (3). Diagnosis is challenging, particularly in younger patients, who often remain asymptomatic despite significant circulatory impairment.

A multimodal approach for follow-up is fundamental as described in detail in Table 1. Trans-thoracic echocardiography (ETT) is a diagnostic cornerstone and should be performed at least yearly (12).

**Table 1:** Proposed organ system surveillance strategy.

**Organ system**	**Exam type**	**Timing**	**About**
Cardiovascular	Clinical evaluation	6–12 months	Depending on clinical condition
	ECG and ETT	6–12 months	ETT should include measurement of VCI diameter, TAPSE/MAPSE, Simpson/FAC, E/A, E/E', doppler of pulmonary veins and GLS
	ECG-Holter	Every 1–3 years	Depending on time from surgery and clinical condition. Frequency should be intensified in time considering the increased risk of arrhitmias
	Exercise stress test	Every 1–3 years	Depending upon the age of the patient to identify changes in exercise capacity, arrhythmias, or desaturation with exercise that may prompt further evaluation
	Serum BNP/NTproBNP and TpI	Every 1–2 years	Depending on clinical condition, previous levels and timing of other blood tests
	Cardiac MRI	Every 2–3 years	Depending on clinical condition and previous findings
	CT angiography	As clinically indicated	
	Cardiac catheterization	As clinically indicated	At least every 10 years according to AHA
Liver	Clinical evaluation	As clinically indicated	Consultation with a hepatologist experienced in hepatocardiac disorders and Fontan patient is recommended in case of evidence of liver disease
	Liver function blood tests (AST, ALT, GGT, PT, PTT, lipidic profile, ALP)	Every year	Depending on clinical condition
	α-fetoprotein	Every year	In case of cirrhosis, alphaFP should be detected every 6 mo for HCC screening
	HCV and HBV sierology	At least once	
	Abdominal US	At least every 2 years	US should investigate the liver, spleen parenchyma morphology (echo structure and echogenicity), to reveal any signs of portal hypertension (i.e., measuring spleen size, porta vein diameter and portal flow velocity rate) or systemic venous hypertension [i.e., measuring inferior vein cava (IVC) and hepatic veins diameter]
	Fibroscan	At least every 5 years	In case of liver dysfunction, the exam has to be anticipated
	EGDS	As clinically indicated	In case of cirrhosis, EGDS shoud be performed every year to monitor esophageal variceal status
	Liver biopsy	As clinically indicated	Liver biopsy should be reserved to patients with focal lesions or in whom a diagnosis of cirrhosis remains in doubt
Lymphatic	Serum albumin level, protein profile	Every year	
	Serum IgG	Every 1–4 years	Depending on level of suspicion for PLE (levels of serum albumin, total protein and absolute lymphocyte count)
	Fecal alpha-1 antitripsin	Every year	The detection should be done on a 24-hour stool sample. Increased 24-h clearance of alpha-1-antitrypsin is suggestive of PLE
	Oxygen saturation	At each clinical control	Routine surveillance of oxygen saturations may promote early detection of plastic bronchitis
	Chest X-ray	Every year	Useful to monitor heart size and pulmonary vascularity. Pleural effusions may suggest PLE or other hemodynamic abnormality
	Lymphatic MRI	As clinically indicated	Depending on center experience
Renal	Clinical evaluation	As clinically indicated	Consultation with a nephrologist experienced in Fontan Fontan patient is recommended in case of evidence of kidney disease
	Blood test (creatinine with GFR, urea, cystatin-C, sodium, potassium, cloride, calcium, phosphate, PACE)	Every year	
	Urine standard exam (proteinuria, albumin/creatinine ratio)	Every 1–2 year	
	24 h urine sample tests (NGAL, NAG, proteinuria, microalbuminuria)	Every year	
	Renal US with doppler	Every year	Renal US should include evaluation of renal diameters and echogenicity and doppler evaluation of RRI (renal resistance index) to estimate vascular compliance
Other exams	Full blood count	Every year	
	Metabolic profile	Every year	
	Vitamine D and PTH	Every year	
	Endocrine/metabolic profile	Every year	
	Ammonium	Every year	
	Iron metabolism	Every year	

The complex anatomy and lack of normal reference values and functional indexes render the quantification of cardiac function problematic (12). The introduction of 3D ETT has provided useful information on the structure of valve leaflets and subvalvular apparatus. In patients with poor acoustic windows, transesophageal ETT can provide excellent and more detailed imaging. Cardiac MR (CMR) helps to characterize ventricle performance, valvular function, and flow data (13). CT is an excellent alternative to CMR. Holter-ECG monitoring, exercise stress tests, and annual laboratory tests are useful to track each patient's individual path and to guide treatment (3, 14). With regards to the failing Fontan, the first approach should aim to ameliorate anatomical defects (i.e., pulmonary venous obstruction, pulmonary artery stenosis, obstruction within the Fontan baffle, or aorto-pulmonary collaterals) by surgical or interventional strategies (3, 15). For example, the timing of the surgical approach to AVV regurgitation should be individualized: on the one hand, a delayed intervention can cause irreversible damage to the pulmonary system and ventricular function, on the other side, a premature intervention may cause surgical failure or postoperative valve regurgitation (16).

Pharmacological therapy for HF has not been established in adult patients with Fontan but it is reasonable to treat SV dysfunction with standard medication used in biventricular HF (3, 7). The use of angiotensin-converting enzyme (ACE) inhibitors has shown conflicting results in patients with Fontan (17, 18); beta-blocking agents might be helpful to reduce the sympathetic hyperactivity responsible for myocardial hypertrophy, but may also increase the risk of cardiac sinus block. As of today, there is still no clear data about the benefits of nesiridite but it is believed to be an hypothetically interesting therapeutic agents in acute decompensated HF (19). Phosphodiesterase inhibitors and endothelin receptor antagonists may be used to decrease vascular resistance and improve preload to treat pulmonary hypertension (20). In diastolic HF, diuretic therapy is the only medication proven to be effective. Less common treatments include cardiac resynchronization therapy, fenestration creation, mechanical circulatory support, and conversion of an atriopulmonary Fontan connection to an extracardiac conduit (ECC) Fontan (7, 21). Although the improvement in recent Fontan survival is impressive, it remains a palliative procedure, the only curative treatment remains heart transplantation, especially in those with major Fontan-associated morbidities. Transplant indications, contraindications, and the optimal timing of intervention are still debated but outcomes in children look promising (22).

### Arrhythmias and thromboembolism

The anatomical changes brought by the subversion of the heart anatomy lead to a predisposition to arrhythmias, which are an important contributor to morbidity. Furthermore, arrhythmias can increase the risk of thromboembolic events (TEs), which have a mortality rate of 25% (23, 24). On the other hand, all patients with Fontan have *per se* a higher risk of TE related to the low-flow state, atrial stasis, and increased central venous pressure; in particular, the latter causes hepatic dysfunction and therefore coagulation abnormalities (23, 25). The incidence of arrhythmias after Fontan operation increases with age and time from surgery, and it is estimated to be around 25%−60% (26). Tachyarrhythmias have the highest prevalence, the most common being intra-atrial reentrant tachycardia (IART) (3, 26–28), while sinus node dysfunction is the most frequent bradyarrhythmia (3, 26). Several factors contribute to the development of atrial dysrhythmias, such as manipulation of the atrial wall with possible injury to the sinus node during surgery or atrial dilation and hypertrophy following modification of the hemodynamic status. The type of surgery plays another important role, in particular, atriopulmonary connection is the surgical technique associated with the greater risk of developing both atrial arrhythmias and TEs (27–30). Between ECC and lateral tunnel, studies suggest that the first is associated with a minor risk of arrhythmias (3, 7, 28, 29), probably having a minor impact of the atrial tissue (28). Early detection of arrhythmias in patients with Fontan by a regular cardiological follow-up, such as transthoracic echocardiogram (TTE), ECG, Holter-ECG, and blood tests, is crucial to both prevent thrombosis and manage hemodynamic consequences (Table 1). Patients at high suspicion for TE deserve supplementary exams, such as TEE, CT, and CMR (25). Antiarrhythmic drugs in Fontan must be used with caution, especially inotropic negative ones, because these can critically alter ventricular contractility and vascular resistance (1, 3, 31). Moreover, in patients with an atriopulmonary connection, a conversion to an ECC Fontan has been shown to improve arrhythmia outcomes and hemodynamics (3, 31, 32). When recognized promptly, IART can be treated by direct current cardioversion (31, 33). Pacemaker implantation is indicated if significant sinoatrial node dysfunction is present (30, 31). According to most studies, there is a lower incidence of TE in patients receiving prophylaxis when compared with no TE-prophylaxis. The current American College of Chest Physicians (CHEST) guidelines recommend the use of antithrombotic therapy, either aspirin or warfarin, as medical prophylaxis in patients with Fontan. However, the agent of choice and the optimal duration of treatment remain controversial (7, 23, 25, 32). Data comparing warfarin and aspirin showed similar outcomes, and, at the moment, there is a paucity of safety and efficacy evidence on the use of direct oral anticoagulants in patients with Fontan (33). In current practice, aspirin can be used as the primary prophylactic agent and the introduction of warfarin, with a goal international nor-malized ratio (INR) of 2–3, can be considered in patients with risk factors, but also taking the possible bleeding risk on account (29, 31, 34). In a recent retrospective study evaluating the incidence of late clinical TE in a large cohort of patients with Fontan after different prophylaxis strategies, warfarin showed no better outcomes when compared with aspirin, despite a higher combined rate of major and clinically relevant bleeding. These findings suggest the possibility of a simpler antithrombotic regimen, for example, starting with warfarin in the first 3–6 months, then switching to aspirin (32). At the moment, no risk stratification scores for TE are available for patients with Fontan and the standard risk stratification scores, such as CHADS2/CHADS2-VASc scores, seem to be not predictive in patients with congenital heart disease, as evidenced in a relevant multicenter study (24, 34). More studies are needed to develop a risk stratification tool to guide antithrombotic therapy and to compare the different treatment strategies.

### Veno-lymphatic complications

The higher central venous pressure and the lower cardiac output of the Fontan circulation obligate the lymphatic system to operate at functional limits causing lymphatic overproduction, creation of abnormal channels, and lymphatic hypertension. This can be tolerated for years, but in the long term, it determines leakage of the fluids in the interstitium, and complications related to lymphatic insufficiency will occur. Every anatomical district can be affected, but the most relevant includes protein-losing enteropathy (PLE) and plastic bronchitis (PB). PLE is caused by intestinal congestion due to lymphatic insufficiency and portal hypertension; this leads to enteric protein loss. PB is a disease defined by the presence of thick tenacious casts within the airways secondary to leakage of lymphatic fluid caused by fistulous connections of lymphatic vessels to the airways. Airway inflammatory reaction contributes to pathogenesis. PLE affects 5%−12% of patients with Fontan while PB occurs in <5%. The onset can be 1 month to nearly two decades after the Fontan procedure, usually occurring between 2 and 3 years following the palliation (3). Clinically, PB presents with chronic cough or coughing fits, breathlessness, wheezing, and other respiratory symptoms. Low oxygen saturation and asphyxia may occur because of ventilation-perfusion mismatch. Life-threatening events may occur in up to 40% of patients (35). PLE manifests with edema and ascites due to the reduction of the oncotic pressure. Excessive protein loss can cause diarrhea and fat malabsorption, immunodeficiency, bone abnormalities due to hypocalcemia, and potential gastrointestinal bleeding secondary to altered coagulation (36). The gold standard test to diagnose PLE is an increased α-1 antitrypsin clearance in a 24-h stool collection. Getting the adequate specimen samples is challenging, therefore, the diagnosis can also be made on an increased α-1 antitrypsin level in a single stool sample associated with hypoalbuminemia and edema, with no other identified cause (17). On the other side, diagnosis of PB can be confirmed in the presence of casts by the patient's expectoration or by bronchoscopic removal. Surveillance of lymphatic complications is a relatively new concept so precise guidelines are not available; a suggested follow-up approach is indicated in Table 1. Routine surveillance of oxygen saturation, chest X-ray, and pulmonary function testing are the best options to evaluate pulmonary status. MR lymphography based on heavily T2-weighted sequences can visualize the vascular architecture and eventually map abnormal networks in the chest (24) and, when performed in conjunction with endoscopy, can show leakage into the bowels (37). The goal of treatment, both in PLE and PB, is to optimize systemic circulation and reduce the overload with diuretic therapy. Treatment options for PB include diuretics and pulmonary vasodilator, such as endothelin receptor antagonist (ERAs) and phosphodiesterase type 5 inhibitor (PDE-5) inhibitors, that can be considered in cases of increased pulmonary pressure in selected patients with controlled ventricular end-diastolic pressure (7, 38). Some patients may benefit from anti-inflammatory measures using inhaled or systemic steroids; mucolytics and nebulized tissue plasminogen activators can improve cast expectoration, and bronchodilators and chest physiotherapy can be useful. In PLE, to replace protein loss, intravenous albumin and immunoglobulins can be necessary; dietary modifications can adjuvate the process. Considering the inflammatory patterns, oral controlled-release budesonide may be helpful (20). In some cases, anticoagulant therapy with heparin can help and octreotide may be effective in some patients by decreasing thoracic duct lymphatic drainage (39). Lymphatic intervention, by accessing and occluding the abnormal connection, with selective embolization, is an emerging strategy, potentially promising but still needs technical refinements (40, 41). Currently, these invasive therapies are considered only in selected patients and the only effective cure for lymphatic dysfunction is heart transplantation.

### Liver disease

Hepatic anomalies, known as Fontan-associated liver disease (FALD), constitute an early finding within the first 5 years since the Fontan completion (42, 43). The increased venous pressure in the hepatic veins and sinusoids leads to a decreased portal venous inflow, resulting in progressive liver hypoperfusion; the consequent injury is centrilobular fibrosis up to cirrhosis, with increased risk of hepatocellular carcinoma (44). Clinical manifestations are those of portal hypertension. When considering laboratory markers, liver enzymes and clotting parameters may result in normal even in very advanced stages of hepatic fibrosis. Hyperbilirubinemia generally can be identified only in decompensated liver cirrhosis. Thrombocytopenia, instead, is a useful marker of portal hypertension and correlates with the degree of portal fibrosis (45). Alpha-fetoprotein should be monitored as a predictor of hepatocarcinoma. Follow-up with abdominal imaging, clinical evaluations, and biomarkers is important (7); timing of surveillance should be individualized according to the grade of liver damage (Table 1) (3). Although liver ultrasound is not a sensitive tool for the identification of liver fibrosis, Doppler ultrasound tracing can be useful in the evaluation of portal flow, arterialization of hepatic flow, and changes in celiac and mesenteric flow resistances (3). Ultrasound elastography can provide a non-invasive estimate of the onset and progression of liver fibrosis by evaluation of hepatic stiffness, even though further studies for the standardization of fibroscan scores are needed (46). CT and MR provide accurate information about liver morphology, structure, and vascular enhancement patterns, especially in the characterization of hepatic nodules and hepatocellular carcinoma (47). Liver biopsy remains the gold standard for the evaluation and grading of liver fibrosis but should be reserved for patients with focal lesions or in whom a diagnosis of cirrhosis remains in doubt (46, 47). In terms of treatment, efforts should focus on the prevention of FALD with proper optimization of the Fontan circulation. Patients should be encouraged to maintain a healthy lifestyle and to avoid alcohol and excessive use of acetaminophen; immunization for hepatitis A virus (HAV) and hepatitis A virus (HBV) should be recommended (45). Some promising medical therapies may be of potential benefit but are yet of unproven efficacy in the prevention and stabilization of FALD; these include pulmonary vasodilator therapy, vascular decongestant agents, or antifibrotic therapies (3). Isolated liver transplant is not considered a feasible option, in whom graft cirrhosis would soon develop, therefore, patients with decompensated liver cirrhosis should be carefully selected for combined heart and liver transplant (48).

### Kidney dysfunction

Fontan renal dysfunction can be detected in at least 10% of younger patients and its prevalence increases as they become adults. The decreased cardiac output and the increased central venous pressure (CVP) following Fontan reduce glomerular filtration rate (GFR), and increase glomerular filtration pressure causing glomerular injury (49). The multiple surgeries and the exposure to nephrotoxic medications, iodinated contrast agents, and chronic hypoxia contribute to kidney impairment (3, 50). Monitoring for a renal function is sensible in Fontan patients (Table 1). Proteinuria and microalbuminuria are useful markers, and cystatin C-based GFR gives a more accurate assessment of renal function as compared to creatinine (51). Recent evidence showed that ETT can be useful to predict the risk of nephropathy by monitoring the indexed inferior vena cava diameter (iIVC) as a surrogate for high CVP if enlarged (52). Furthermore, measurement of the renal resistive index (RRI) is important to estimate vascular compliance. An increased RRI (>0.81) detects progressive renal injury related to HF and increased mortality (53). Measures that preserve kidney function include hydration, blood pressure control, and saving of toxic drugs; it is unclear if medications, such as ACE inhibitors, have a protective renal effect (3, 49).

## Surveillance strategy of patients with Fontan and the role of a multidisciplinary approach

The survival rate of patients with Fontan is increasing, and these children require lifelong follow-up with medical care and management of their multiorgan complications. As patients with Fontan move into adulthood, there is a progression of organ system complications and therefore, the optimal surveillance strategy should be tailored to each patient, considering age, stage of surgery, and general clinical conditions. It is reasonable to offer surveillance testing that systematically evaluates cardiovascular and end-organ health periodically; however, at this moment, there is insufficient evidence to precisely define a universal timing and panel of tests for the surveillance strategy of patients with Fontan. We therefore suggest a scheme to guide follow-up (Table 1), according to the surveillance testing toolkit proposed by the American Heart Association (AHA) writing group and that is based most on our current practice. The main goal of the Fontan surveillance strategy is to include a multidisciplinary team to approach not only cardiovascular but also end-organ complication with caution and to adapt any intervention to each patient's life stage, development, and needs. Future studies are needed to better understand the progressive nature of end-organ dysfunction in Fontan circulation and to evaluate further strategies for surveillance and treatment of complications.

## Author contributions

DM collected the studies available in literature about arrhythmias and thromboembolism in Fontan circulation. CR studied about heart failure. FT studied about lymphatic complications. GSt studied about the liver and implemented the bibliography. CG collected information about renal and neurological complications. SV provided the introduction. CR and DM summarized all the information and created a model of follow-up based on a multidisciplinary approach with the table. SV, CG, and FT arranged the lay out. All authors conducted a literature research with key words such as “Fontan complications”, “long term complications” and it was conducted separately for each complication in order to find the majority of available studies. They reviewed the articles and finally provided a scheme with the recent evidence about the correct diagnosis and management of this problem.

## Conflict of interest

The authors declare that the research was conducted in the absence of any commercial or financial relationships that could be construed as a potential conflict of interest.

## Publisher's note

All claims expressed in this article are solely those of the authors and do not necessarily represent those of their affiliated organizations, or those of the publisher, the editors and the reviewers. Any product that may be evaluated in this article, or claim that may be made by its manufacturer, is not guaranteed or endorsed by the publisher.

## Abbreviations

AVV: atrio-ventricular valve
CVP: central venous pressure
ECC: extra-cardiac conduit
FALD: Fontan-associated liver disease
HF: heart failure
PB: plastic bronchitis
PLE: protein-losing enteropathy
TE: thromboembolic events.
